# Combination of electrophysiological mapping, radiofrequency catheter ablation, and atrial appendectomy in a 5-year-old girl with tachycardia-induced cardiomyopathy: a case report

**DOI:** 10.1186/s13019-024-02693-z

**Published:** 2024-04-02

**Authors:** Min Zhang, Xiaoxiao Cao, Yong Zhang

**Affiliations:** Wuhan Women and Children Medical care center, Tongji Medical College, Wuhan Children’s Hospital, Huazhong University of Science & Technology, 100 Hongkong Road, Jiangan District, Wuhan, Hubei China

**Keywords:** Tachycardia-induced cardiomyopathy, Atrial appendectomy, Radiofrequency catheter ablation, Atrial tachycardia, Electrophysiological mapping

## Abstract

**Background:**

Atrial tachycardia (AT) originating from the left atrial appendage (LAA) is uncommon and the most difficult arrhythmia to eliminate. Therefore, we present the case of a 5-year-old girl with tachycardia-induced cardiomyopathy (TIC) caused by AT originating from the LAA and successfully treated with RFCA associated to left atrial appendectomy. With resolution of AT, we observed a progressive improvement of LV function. The effectiveness and safety of this combination therapy were evaluated over a one-month follow-up period.

**Case presentation:**

A 5 -year-old female was evaluated for three days of incessant cough and a syncopal episode. Surface echocardiography and 24-hour monitoring showed that the infant had persistent atrial tachycardia. Echocardiography revealed an enlarged tele diastolic diameter (46.1 mm) and malfunctioning (EF 28.53%) left ventricle. The location of the lesion at the apex of the LAA was further confirmed by electrophysiological study and RFCA. After RFCA, the infant’s ECG monitor showed that sinus rhythm was maintained for up to 22 h. Subsequently, atrial tachycardia recurred and sinus rhythm disappeared. Finally, atrial appendectomy was performed and sinus rhythm returned to normal.

**Conclusions:**

The heart function of the infant improved and sinus rhythm was maintained, further demonstrating the safety and effectiveness of combined treatment with RFCA and atrial appendectomy after electrophysiological localization of AT from LAA to TIC.

## Background

Atrial tachycardia (AT) originating from the left atrial appendage (LAA) is rare and the most difficult to eliminate due to its location [1]. ATs can easily develop tachycardia-induced cardiomyopathy (TIC), which is defined as the reversible impairment of ventricular function due to persistent arrhythmia [2, 3]. Ectopic ATs are unlikely to resolve spontaneously, and antiarrhythmic drugs such as propafenone and amiodarone are usually ineffective. Early use of radiofrequency catheter ablation (RFCA) is recommended to cure patients. Therefore, in our report, we present a case of the 5-year-old girl with tachycardia-induced cardiomyopathy caused by AT originating from the LAA and describe that the arrhythmia was successfully treated with RFCA associated to left atrial appendectomy. With resolution of AT, we observed progressive improvement in LV function.

### Case presentation

**Fig. 1:**
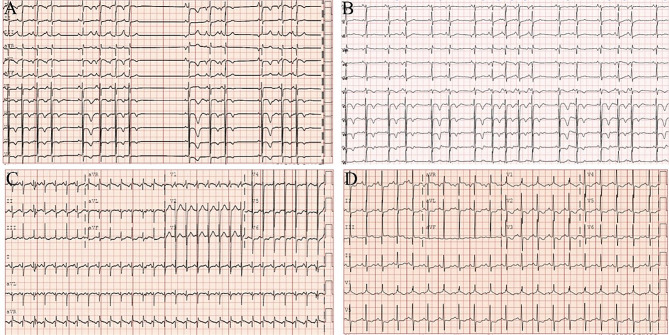
The change of ECG before treatment (**A**), after radiofrequency catheter ablation (RFCA) (**B**), after combined RFCA with atrial appendectomy (**C**), and after 1-month follow-up (**D**). (The paper speed of all electrocardiograms is 25 mm/s and the basic calibration voltage is 10 mm/mV)

A 5-year-old female infant (weight 23 kg and height 120 cm) was evaluated in Wuhan Children’s Hospital for three days of incessant cough and a syncopal episode. Three years ago, the child suffered from heart palpitations after contracting an acute upper respiratory tract infection. A year ago she had a brief syncope that was accompanied by pallor. However, the parents were neither examined nor treated for the cause of the illness at this time. During this hospitalization in our hospital, physical examination showed that the infant’s heart rate was 160 beats per minute, and arrhythmias were noted. Furthermore, the infant’s heart sound was slightly low and no obvious heart murmurs were heard. The infant’s respiration was 30 fractions and palpation of the liver was normal. CK-MB and hypersensitive troponin T were normal values. 24 h Holter monitoring revealed sustained atrial tachycardia (Fig. 1a) with eight sinus stops lasting more than 2 s, with the longest RR interval of 2265 s and an average heart rate of 149 beats per minute. Echocardiography revealed an enlarged tele diastolic diameter (46.1 mm) and malfunctioning (EF 28%) left ventricle.

**Fig. 2:**
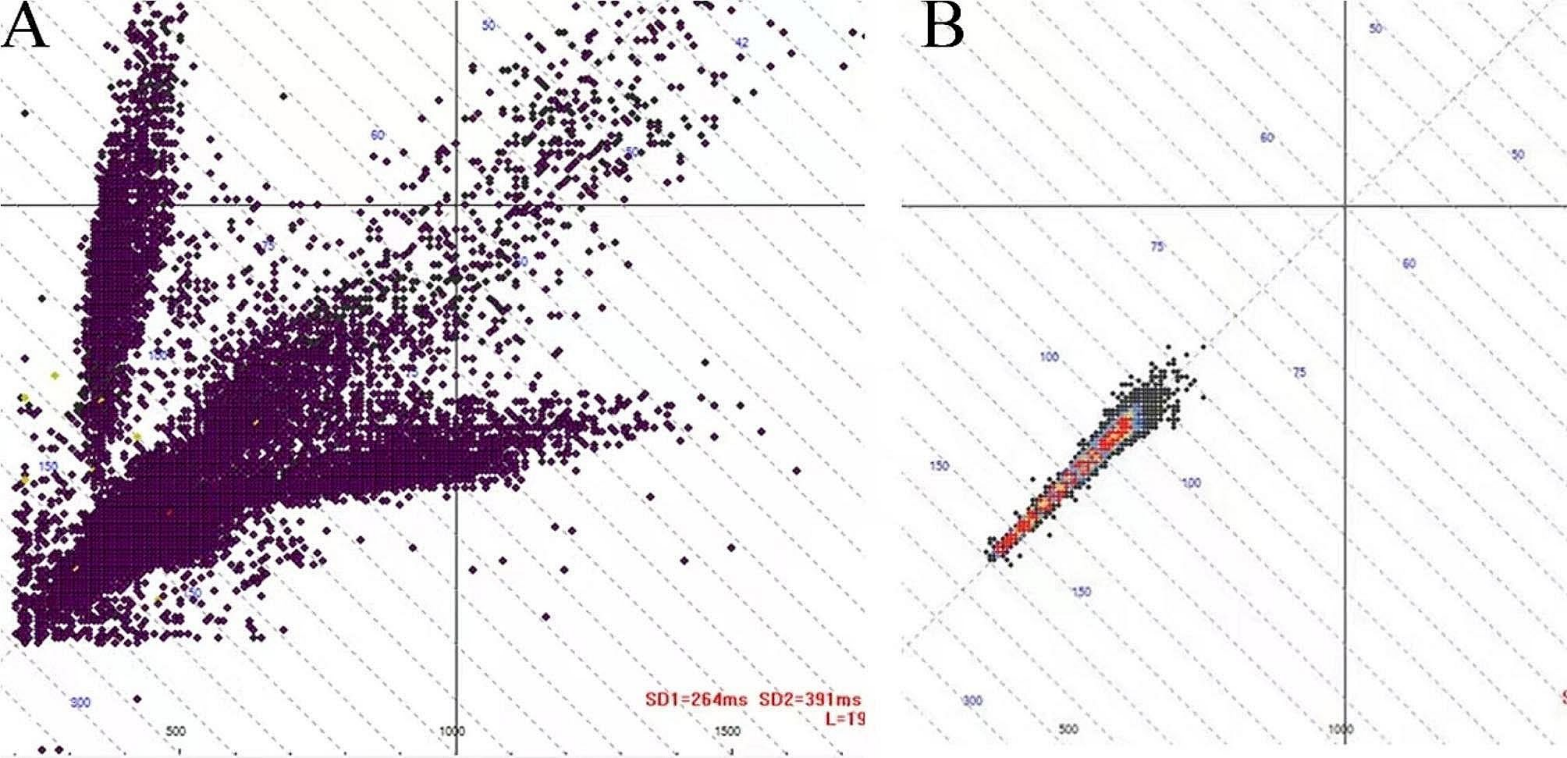
Scatterplot of 24-hour electrocardiogram before combined treatment of RFCA and atrial appendectomy (**A**). Scatterplot of 24-hour electrocardiogram after combined treatment of RFCA and atrial appendectomy (**B**)

Intravenous injection of deslanoside was ineffective in rate control and remained uncontrolled at 160 beats per minute (Fig. 2a). Due to the poor cardiac function of the infant, RFCA under general anesthesia was considered after obtaining informed consent [2, 4, 5]. The infant had persistent tachycardia since RFCA. Two venous accesses were created. A 6 F-decapolar and a 6 F-quadripolar catheter were advanced into the coronary sinus and right ventricular apex via the left subclavian vein and right femoral vein, respectively. The ablation catheter was advanced into the left atrium using a transseptal puncture technique because the foramen ovale had closed.

**Fig. 3:**
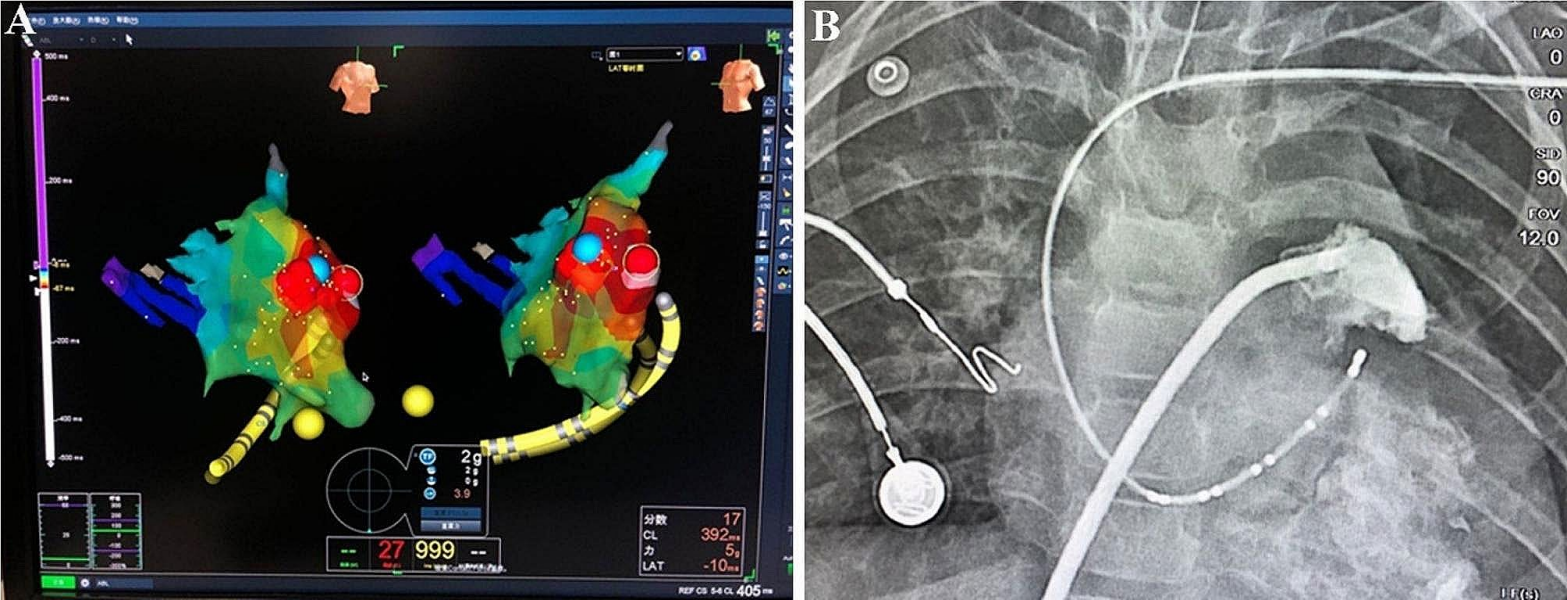
Three-dimensional electroanatomical mapping (**A**) and the angiogram (**B**) of left atrial appendage (LAA). **Panel A**: early electrograms were depicted in red and red dots were the sites at which ablations were attempted; **Panel B**: The ablation catheter successfully entered the left atrial appendage. The LAA and left atrium can be seen at the right anterior oblique angle of 30 degrees (left part) and the light anterior oblique angle of 30 degrees (right part)

Three-dimensional electro-anatomical mapping systems (St. Jude Medical, St. Paul, MN, USA) were used to map tachycardia at the right anterior oblique angle of 30 degrees (RAO 30°) and the light anterior oblique angle of 30 degrees (LAO 30°). Mapping near the LAA showed early signals in the LAA area. The isochronous activation map of tachycardia was constructed based on the proximal atrial CS signal to show that the lesion was located at the distal end (apex) of the LAA (Fig. 3a). A 4-mm open-irrigated-tip catheter (St. Jude Medical, St. Paul, MN, USA) was used at the site. Over the course of a 3-hour treatment, radiofrequency energy was delivered to five lesions within 40 s at a target temperature of 43 °C, a power of 20–30 W and a flow rate of 17 ml/min. AT stopped during RFCA, indicating that the lesion was in the left atrial appendage and confirming that AT was spontaneous and focal. After the AT stopped, sinus rhythm occurred, but it recurred a few seconds later, forcing us to consider further treatment options. This could be reversed to show that atrial tachycardia in the infant was spontaneous and focal.

**Fig. 4:**
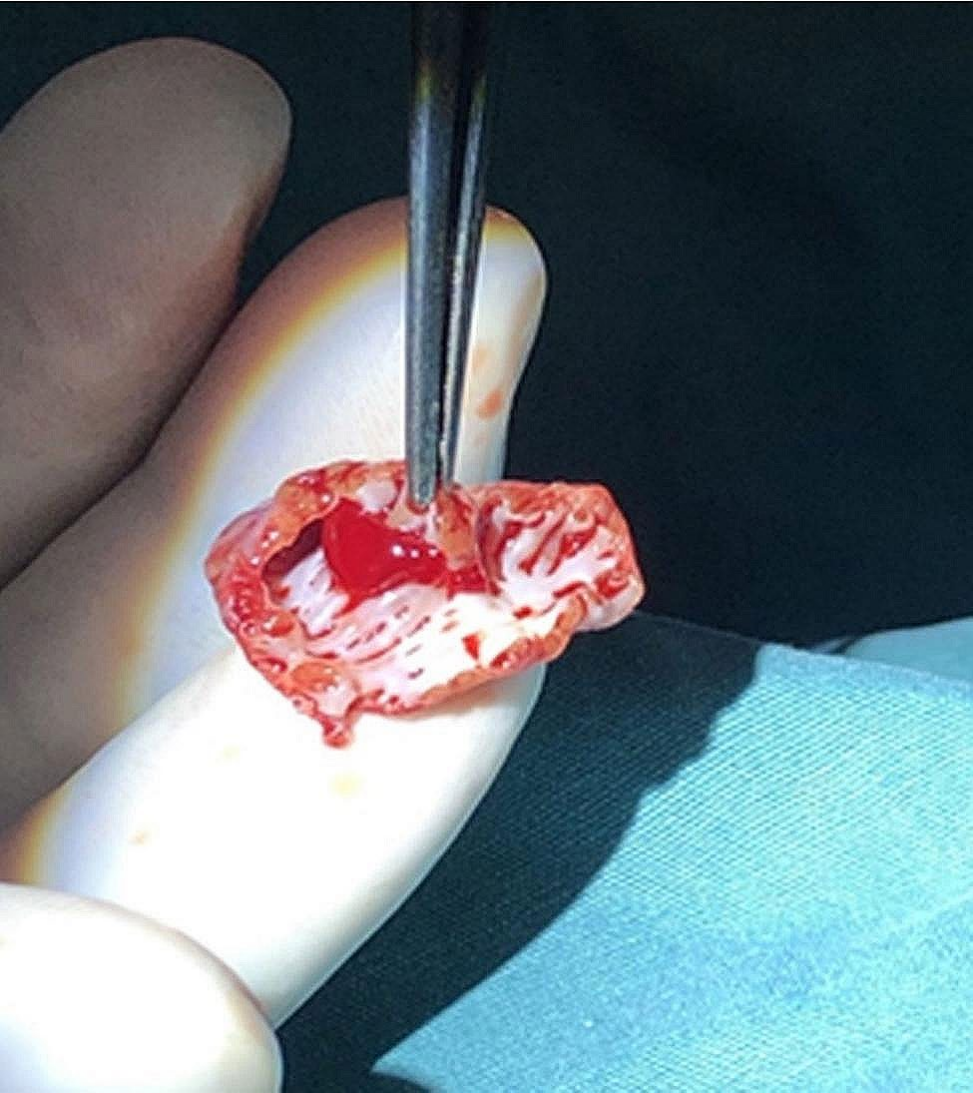
Surgical images of the left atrial appendage reveal a complex anatomical structure consisting of a thick pectinate muscle band and a thin-walled tissue

Due to the high risk of perforation in the LAA, LAA resection was performed with parental consent on the second day after radiofrequency ablation. Based on the precise location of the lesion in the LAA identified by electrophysiological mapping and RFCA, the infant’s chest was opened through a left posterolateral incision under general anesthesia. The LAA was partially detached from the atrium using a clip at the base, and then a left atrial appendectomy was performed (Fig. 4). As the procedure progressed, AT ended after LAA resection and sinus rhythm, with an appropriate frequency profile being established immediately (Figs. 1c, 2b). The echocardiogram showed that LVDd decreased from 46.1 mm to 37.1 mm, while LVEF increased from 28.53 to 46.2% (Table 1). The infant remained asymptomatic in sinus rhythm after a 1-month follow-up (Fig. 1d).

**Table 1:** Echocardiography parameters before and after the therapy

	LVDd (mm)	LVDs (mm)	LVEF (%)	Cardiothoracic ratio
Before therapy	46.1	39.5	28.53	0.64
2 days after therapy	40.1	36	33	/
6 days after therapy	39.72	32.08	40	0.53
1 month after therapy	37.1	33.4	46.2	/

LVDd, left ventricular *end-*diastolic diameter; LVDs, left ventricular end-systolic diameter; LVEF, left ventricular ejection fraction

## Discussion and conclusions

Cardiovascular causes result from either blood flow obstruction, myocardial dysfunction, or arrhythmias. Cardiac syncope accounts for approximately 10% of syncope observed in children and young adults [6]. Cardiovascular causes result from either blood flow obstruction, myocardial dysfunction, or arrhythmias, Therefore, heart failure and syncope may be manifestations of chronic atrial tachycardia, especially in pediatric age [7]. . In our case, the infant had syncope with abnormal echocardiogram and ECG findings. Based on these findings, it was hypothesized that TIC caused by incessant ATs originating from the LAA primarily caused syncope in the infant. Finally, the infant in this case was successfully cured by a combination of RFCA and left atrial appendectomy.

TIC is a form of reversible myocardial dysfunction caused by chronic arrhythmias and characterized by ventricular systolic dysfunction, dilatation, and heart failure, which can be reversed by normalizing the heart rate [3, 8]. The pathological mechanism of atrial TIC is mainly cardiomyocyte changes, abnormal energy metabolism and Ca^2+^ processing disorder. Oxidation-reduction reaction stress and the activation of the neurohumoral system also play a role. Incessant ATs can lead to TIC in both adults and children, accounting for 5% of all TIC cases in adults and 14% in children [9]. Appendage tachycardia is characterized by persistence and refractory antiarrhythmic therapy and accounts for 25–50% of all Ats cases, of which 25% develop cardiomyopathy. The infant in our study was considered to have TIC due to extremely low LVEF, enlarged left ventricle, and persistent tachycardia on ECG. Meanwhile, LVEF improved after heart rate normalization [3].

ATs derived from the LAA exhibit typical ECG and electrophysiological features that are helpful in RFCA guidance [10, 11]. The ECG of the patient could predict ATs originating from the LAA and showing negative P waves in leads I, aVL, and positive P waves in leads II, III, and aVF, as well as a positive P wave with M-type in lead V1. Electrophysiological mapping could identify ATs originating from the LAA based on analysis of both the atrial activation sequence and the earliest atrial activation within the coronary sinus relative to the P wave.

ATs have been reported to be refractory to RFCA, with a success rate of approximately 70% [1]; however, the success rate of RFCA in LAA was only about 10% [12]. This is consistent with the results of our case, which suggest that RFCA is effective for ATs originating from the LAA but does not completely eliminate them.

LAA anatomy and electrophysiological parameters are the main reasons for refractory RFCA. The LAA has a special anatomical structure and is the remnant of the embryonic left atrium that develops in the third week of pregnancy. The anatomical structure of the LAA is very complex and consists of a thick pectinate muscle band and a thin-walled tissue [13, 14]; T Therefore, it has been reported to be a focus for ATs [10, 15–17]. In venography, the appendage consists on average of three sectors: the base, the middle and the apex [13]. There are three reasons why it is difficult to eliminate atrial tachycardia in LAA by radiofrequency ablation: (1) The ablation catheter cannot accurately reach the origin site of the atrial tachycardia due to the influence of the pectinate muscle; (2) The wall of the left atrial appendage is thin and prone to myocardial perforation. (3) The RF energy is influenced by the pectinate muscle and cannot reach the energy to eliminate the local ectopic focus, especially in the distant area (or tip) of the LAA, a rare site of origin of the focal AT (2.1%) [18]. In our case, the infant was treated with a combination of RFCA and auricular appendectomy, which improved cardiac function and maintained sinus rhythm, further demonstrating the safety and effectiveness of combination therapy after electrophysiological localization of AT from LAA to TIC.

The combination treatment of TIC in children with persistent AT from LAA consists of three steps: First, ECG was used to preliminarily determine the origin of AT. Electrophysiological mapping and RFCA were then performed to confirm the exact location of the lesion in the LAA, followed by ablation therapy. Finally, atrial appendectomy becomes the definitive treatment after failure of RFCA, especially for patients with TIC. Electrophysiological mapping and RFCA were particularly important in establishing an accurate diagnosis, determining the precise focus, and terminating the lesion for atrial appendectomy [19].

We have conducted a summary analysis of this case. (1) The child had a history of syncope, but was not further examined and treated by his parents, which led to the occurrence of TIC and the lack of some treatment options. Therefore, it is very important to actively treat and examine children’s physical diseases. (2) Effective ECG interpretation is very important. Diagnosis of AT begins with a routine body surface ECG, which can first determine the cause of the lesion and then repeat a 24-hour ECG to determine the proportion of AT. Determining whether cardiomyopathy is present depends on echocardiography. In summary, doctors can reduce the possibility of misdiagnosis through effective ECG and echocardiography. 3.The main treatment methods for AT include oral medications, endocardial radiofrequency ablation, epicardial radiofrequency ablation, cryoablation, and left atrial appendectomy. Some literature suggests that pericardial radiofrequency ablation and cryoablation could be effectively used for left atrial appendage atrial tachycardia. However, our hospital had no experience with such cases at that time, we did not choose these two operations, which are very worthy of our trial and summary in future clinical work.

The infant in our study diagnosed with TIC had the lowest left ventricular function of all reported cases in children with LAA-AT. The infant was successfully cured by RFCA combined with atrial appendectomy after electrophysiological localization of AT from LAA. After a one-month follow-up, this combined therapeutic strategy was found to be safe and effective. However, the long-term safety and effectiveness of combination therapy in children should be further evaluated.

## Data Availability

The datasets used and analyzed during the current study are available from the corresponding author on reasonable request.

## Abbreviations

AT: Atrial tachycardia
LAA: Left atrial appendage
RFCA: Radiofrequency catheter ablation
TIC: Tachycardia-induced cardiomyopathy
ECG: Electrocardiogram
LVDd: Left ventricular end-diastolic diameter
LVDs: Left ventricular end-systolic diameter
LVEF: Left ventricular ejection fraction
